# Retraction: The intervention of classical and molecular breeding approaches to enhance flooding stress tolerance in soybean – An review

**DOI:** 10.3389/fpls.2024.1441679

**Published:** 2024-06-06

**Authors:** Frontiers Editorial Office

The journal retracts the 2022 article cited above.

Following publication, the publisher found evidence of peer review manipulation. As the scientific integrity of the article cannot be guaranteed, and in adherence to the recommendations of the Committee on Publication Ethics (COPE), the article is retracted.

This retraction was approved by the Chief Executive Editor of Frontiers. The authors received a communication regarding the retraction and had a chance to respond. This communication has been recorded by the publisher.

Frontiers would like to thank Bruce Benson for contacting the journal regarding the published article.

